# Ecological Status of Coralligenous Macroalgal Assemblages in the Marine Protected Area (MPA) Isole Ciclopi (Ionian Sea)

**DOI:** 10.3390/plants10020329

**Published:** 2021-02-09

**Authors:** Luca Giuseppe Costanzo, Giuliana Marletta, Giuseppina Alongi

**Affiliations:** 1 Department of Biological, Geological and Environmental Sciences, University of Catania Via Empedocle, 58-95128 Catania, Italy; luca.costanzo@studium.unict.it (L.G.C.); alongig@unict.it (G.A.); 2 Marine Protected Area “Isole Ciclopi”, Via Dante, 28-95021 Aci Castello, Italy

**Keywords:** coralligenous biocenosis, marine strategy directive, MPA Isole Ciclopi, phytobenthic assemblages, Mediterranean Sea

## Abstract

The coralligenous habitat represents one of the most important hotspots of Mediterranean biodiversity. However, along the Ionian coast of Sicily (Italy) the coralligenous macroalgal assemblages have always been poorly studied. The present study was carried out in the coralligenous habitat of the Marine Protected Area (MPA) Isole Ciclopi, located along the central-eastern coast of Sicily. Previously, only a few floristic studies, including some data on the coralligenous flora, were conducted within this MPA in the 1970s and 2001. Therefore, the present study aimed to gain an updated knowledge on the coralligenous flora and to compare the current data with data derived from the previous floristic studies, to observe if in the last 50 years environmental changes occurred and to monitor the effectiveness of the MPA in protecting this habitat. In particular, the coralligenous flora of the MPA was analyzed through remotely operated vehicles (ROV) surveys and destructive samples. ROV surveys allow us to observe that the coralligenous assemblages of the MPA are well-structured, especially regarding the encrusting Rhodophyta, which showed the highest percent cover among the main morphological groups/taxa. Through the sample analysis in the laboratory, a total of 92 taxa has been recorded. Comparing the floristic lists of the present research and the past studies, an increase of local biodiversity was highlighted. Nevertheless, an increment of Non-Indigenous Species (NIS), warm-water species, and Rhodophyta with wide ecological valence was also noted. The main causes of these variations in the coralligenous flora have been traced back to reduced water transparency, maybe due to sedimentation, and a rise in the seawater temperature. Therefore, although the coralligenous assemblages of the MPA Isole Ciclopi appear to be well-structured, future studies will be necessary to continue monitoring this habitat to evaluate whether the MPA is effective in safeguarding this hotspot of biodiversity.

## 1. Introduction

Coralligenous macroalgal assemblages are a peculiar feature of deep subtidal systems in the Mediterranean Sea [1]. They are characterized by calcareous structures consisting of encrusting Rhodophyta belonging to the orders Corallinales and Hapalidiales, which develop on rocky coasts or sandy planes, under stable conditions of temperature, salinity, and currents and where irradiance is reduced down to 0.05–3% of the surface irradiance [1,2]. The coralligenous habitat represents one of the most important systems in the Mediterranean Sea for its biodiversity, production, and role in the carbon cycle [3,4,5,6,7]. The growth of these bioconstructions depends on a delicate balance between bioconstruction (acted by encrusting red algae, with an accessory contribution by serpulid polychaetes, bryozoans, and scleractinian corals) and bioerosion (performed by borer species, such as molluscs, polychaetes and excavating sponges, and through physical abrasion) [2,8]. In particular, coralline macroalgae of the genera *Mesophyllum*, *Lithophyllum,* and *Neogoniolithon* provide the greatest contribution to coralligenous bioconstructions [1]. However, this balance between building and bioeroding processes can be easily disturbed by environmental changes [2,9]. Indeed, several types of stressors (e.g., sedimentation, biological invasions, and nutrient enrichment) cause severe shifts in the structure of macroalgal coralligenous assemblages [10,11,12,13]. Therefore, stable environmental conditions are essential for the development and survival of coralligenous assemblages [14]. Due to their value, the coralligenous bioconstructions are protected by different international conventions, such as the Habitat Directive 92/43/EEC, the SPA/BIO Protocol, the Barcelona Convention, the Berne Convention and European Union 1967/2006 Regulation [15].

**Figure 1:**
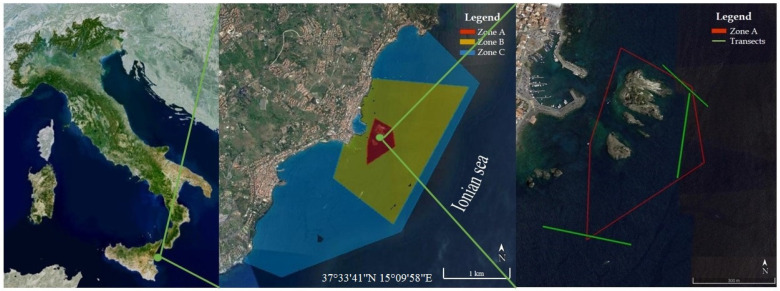
Geographical location of the study area along the Ionian coast of Sicily; location of the Marine Protected Area (MPA) Isole Ciclopi with the indication of the zonation pattern; detail of the zone A showing in green the Remotely Operated Vehicles (ROV) transects (source: Google Earth).

The present study aims to provide updated knowledge on the phytobenthic component of the coralligenous assemblages along the Ionian coast of Sicily (Italy). In particular, this study was carried out on the coralligenous habitat of the Marine Protected Area (MPA) Isole Ciclopi, located along the central-eastern coast of Sicily (Figure 1). This MPA was established in 1989 with the aim to preserve and restore both faunistic and floristic biodiversity, with specific regard to two EU priority habitats: *Posidonia* beds and reefs, this last including the coralligenous. In this area, no study on the coralligenous flora has been performed so far. Some data on the macrophytobenthos of this habitat can be only deduced from floristic studies carried out in the 1970s and 2001, which included some transects in the coralligenous of this MPA [16,17,18]. Therefore, to observe if in the last 50 years there was a variation of the environmental conditions, the current data were compared with data derived from the previous floristic studies realized in the MPA. Indeed, floristic lists are often an important source of botanical information for a particular area and may be used for general comparisons of the vegetation of the same locality at different times [19]. Furthermore, these comparisons can be useful to verify and monitor the effectiveness of the MPA and its role in protecting biodiversity.

## 2. Materials and Methods

The present study was carried out during 2018 through ROV (Remotely Operated Vehicles) surveys and destructive samplings in zone A and the nearby zone B of the MPA Isole Ciclopi (Figure 1). The seabed in this area has a sloping topography, from the coastline to about 40 m depth, and consists of basaltic bedrock locally covered with large volcanic blocks [20]. Three transects, each with an extension of ca. 200 m and in a range of depth of 32–39 m (Table 1), were performed by using a ROV Mariscope, FO II, equipped with a high-definition video camera (GoPro 5) (4 K video resolution, 3840 × 2160 screen resolution), a digital camera with depth sensor and an integrated compass, two laser beams placed 10 cm apart and used as a metric scale for the images and the visual field, and two led strobes of 13,000 lumen. The videos recorded through ROV were analyzed by the editing video software AVS Video Editor. Subsequently, from ROV videos, an average of 20 frames for each transect was randomly selected according to their sharpness. The obtained frames were analyzed to estimate the percent cover, according to the Braun-Blanquet’s scale (5 = >75%; 4 = 75–50%; 3 = 50–25%; 2 = 25–5%; 1 = 5–1%; + = <1%), of the main morphological groups/taxa characterizing the coralligenous habitat [21]. In addition, since ROV frames do not provide information at a specific level, to achieve better knowledge of the coralligenous flora, two samplings were performed through SCUBA diving to a depth of 36 m. The samplings were conducted in two different seasons (spring and autumn) by placing on the substrate a 20 × 20 cm quadrat and scratching the surface containing the phytobenthic component. Subsequently, the samples were stored in a solution of seawater and 90% ethyl alcohol and carried to the Laboratory of Phycology of the University of Catania for the identification of the macroalgal taxa (see Table 2) according to [22,23,24,25]. Therefore, the data obtained by the laboratory analysis were compared with data derived from the previous floristic studies conducted in the MPA and selected according to the sampling site, the depth and the type of flora. Consequently, since the previous studies did not report information on the structure of the macrophytobenthic assemblages, the comparison of the data was based only on the floristic lists. Furthermore, the floristic lists of the past studies [16,17,18] were corrected according to the modern taxonomy and nomenclature for comparison with our present work.

All data were processed through PAST software for Windows, version 4.03, to obtain diversity indices and the Rhodophyceae/Phaeophyceae (R/P) index was calculated. Furthermore, a Bray–Curtis similarity matrix was constructed based on presence/absence data. Since one of the samplings was carried out in 2019 and all data were processed during the same year, the data of the present study will all refer to 2019. The taxonomic validity of the taxa detected in this research was checked according to [22,23,24,25].

## 3. Results

The frames derived from ROV videos were analyzed to evaluate the percent cover of the morphological groups/taxa characterizing the coralligenous habitat of the MPA Isole Ciclopi. The calcareous Rhodophyta showed the highest cover value represented by the grade four (75–50%) of the Braun-Blanquet’s scale. *Peyssonnelia* sp. pl. and erect corticated Rhodophyta presented a cover value corresponding to grade three (50–25%). Algal turf and Non-Indigenous Species (NIS) had a cover value equal to the grade two (25–5%). *Palmophyllum crassum* was present with a cover degree of one (5–1%) in the frames, while *Halimeda tuna* and flattened Rhodophyta with cortication had the lowest value represented by a percent cover <1% of the Braun-Blanquet’s scale (Table 3).

**Figure 2:**
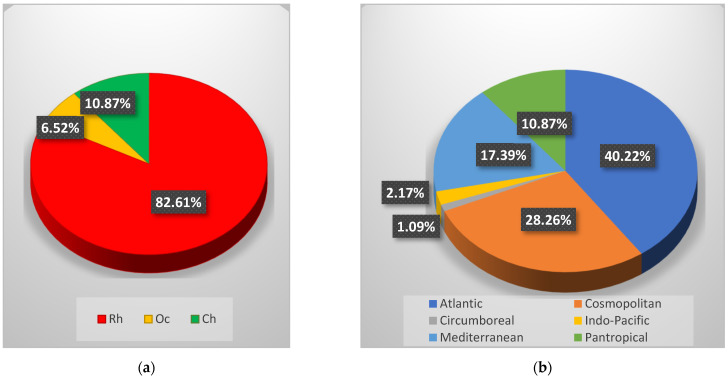
(**a**) Percent incidence of Rhodophyta (Rh), Ochrophyta (Oc), and Chlorophyta (Ch) of the coralligenous flora recorded in 2019; (**b**) Chorological spectrum of the coralligenous macroalgal assemblages recorded in 2019.

Through the sample analysis in the laboratory, a total of 92 taxa (at a specific and interspecific level) (Table 2) was recorded, consisting of 76 Rhodophyta (Rh = 82.61%), six Ochrophyta (Oc = 6.52%) and ten Chlorophyta (Ch = 10.87%) (Figure 2a). Regarding the chorology, the Atlantic was the main phytogeographic element (A = 40.22%), followed by Cosmopolitan (C = 28.26%) and Mediterranean (M = 17.39%) elements. The Pantropical (P = 10.87%), the Indo-Pacific (IP = 2.17%) and the Circumboreal (CB = 1.09%) species were detected with lower percent (Figure 2b).

**Figure 3:**
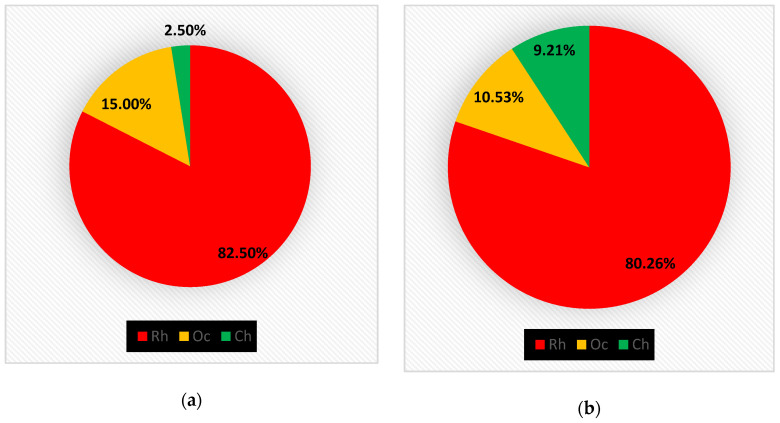
(**a**) Percent incidence of Rhodophyta (Rh), Ochrophyta (Oc), and Chlorophyta (Ch) of the flora studied in the 1970s [16,17]; (**b**) Percent incidence of Rhodophyta (Rh), Ochrophyta (Oc), and Chlorophyta (Ch) of the flora studied in 2001 [18].

**Figure 4:**
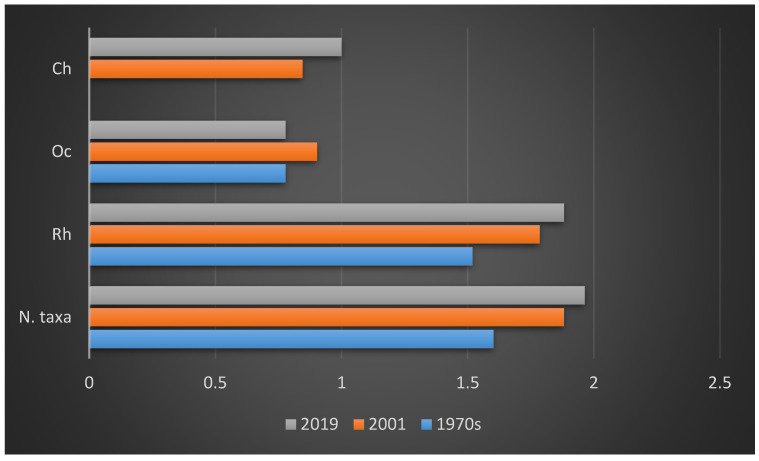
Logarithmic representation of the variations in the number of taxa, Rhodophyta (Rh), Ochrophyta (Oc), and Chlorophyta (Ch) from the 1970s to 2019.

In the 1970s [16,17] 40 taxa were detected, subdivided into 33 Rhodophyta (Rh = 82.50%), six Ochrophyta (Oc = 15%) and one Chlorophyta (Ch = 2.50%), (Figure 3a); while in 2001 [18] 76 taxa were reported, consisting of 61 Rhodophyta (Rh = 80.26%), eight Ochrophyta (Oc = 10.53%), and seven Chlorophyta (Ch = 9.21%) (Figure 3b). Therefore, from the 1970s to 2019, there was an increase in the Rhodophyta and Chlorophyta, while the Ochrophyta remained almost stable. These changes in the coralligenous floras can also be noted by the increase in the R/P index, which from a value of 5.5 in the 1970s passed to 7.6 in 2001 and 12.6 in 2019. Moreover, from the 1970s to 2019 there was an increase in the number of taxa (Figure 4). In particular, in 2001 [18], differently from the previous studies [16,17], 43 species (34 Rhodophyta, three Ochrophyta and six Chlorophyta) were reported, while seven species (six Rhodophyta and one Ochrophyta) were not found anymore. Instead, in the present study, 23 species (20 Rhodophyta and three Chlorophyta), not beforehand reported, were found. However, seven species (six Rhodophyta and one Ochrophyta) have no longer been observed. In the last 50 years, 26 species (22 Rhodophyta, three Ochrophyta and one Chlorophyta) were always recorded in the coralligenous habitat of the MPA (Table 4).

**Figure 5:**
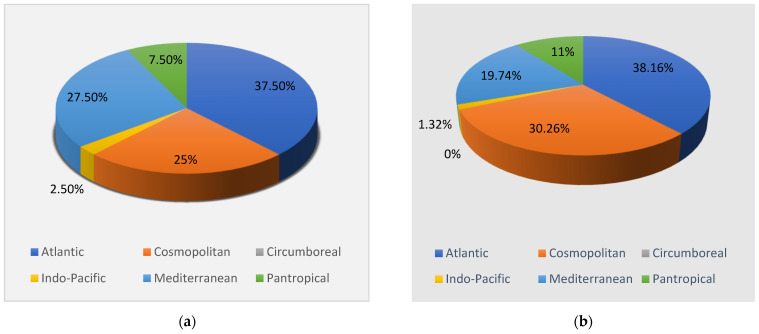
(**a**) Chorology of coralligenous macroalgal assemblages in the 1970s [16,17]; (**b**) Chorology of coralligenous macroalgal assemblages in 2001 [18].

**Figure 6:**
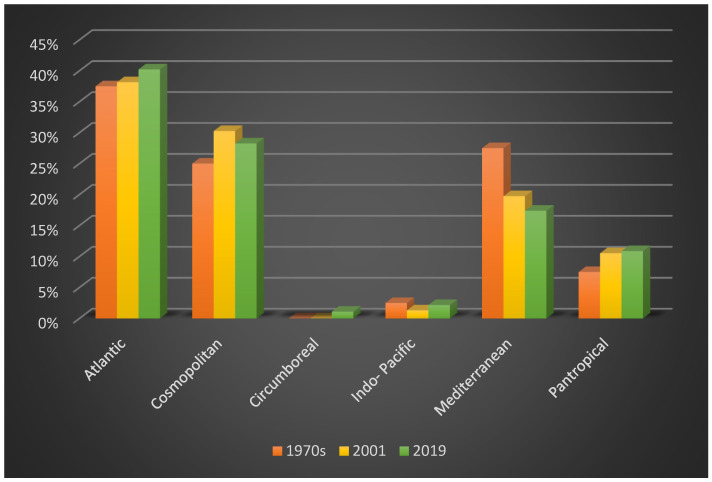
Variations of the percent incidence of Atlantic, Cosmopolitan, Circumboreal, Indo-Pacific, Mediterranean, and Pantropical elements from the 1970s to 2019 in the coralligenous flora of the MPA.

From a biogeographical point of view, in the 1970s, the dominant percent incidence was characterized by the Atlantic (A = 37.5%), Mediterranean (M = 27.5%) and Cosmopolitan (C = 25%) species [16,17] (Figure 5a). In 2001 [18], the highest percent incidence was represented by the Atlantic (A = 38.16%) species, followed by the Cosmopolitan (30.26%) and Mediterranean (19.74%) species (Figure 5b). Consequently, from the 1970s to 2019, there was an increase in Atlantic and Pantropical species, the number of Cosmopolitan and Indo-Pacific species was steady, while the Mediterranean species displayed a strong decline. Only during the present study, a Circumboreal species was found (Figure 6). In particular, of the 43 taxa reported during 2001 [18], 17 were Atlantic, 13 were Cosmopolitan, six were Mediterranean and seven were Pantropical. At the same time in 2001 [18], there was a disappearance of three Atlantic, two Mediterranean and two Pantropical species. In the present study, of the 23 species not previously recorded in this area, nine were Atlantic, five were Cosmopolitan, one Circumboreal, one Indo-Pacific, five Mediterranean and two Pantropical. Moreover, in this study, one Atlantic, two Cosmopolitan and four Mediterranean species, previously present in this area, have not been found anymore. Of the 26 taxa always observed in the coralligenous assemblages of the MPA, there are 11 Atlantic, eight Cosmopolitan, one Indo-Pacific, five Mediterranean and one Pantropical species (Table 5).

Of the 92 taxa found in the present study, five were NIS: *Asparagopsis armata* Harvey, *Lophocladia lallemandii* (Montagne) F. Schmitz, *Anthithamnion amphigeneum* A. Millar, *Bonnemaisonia hamifera* Hariot and *Caulerpa cylindracea* Sonder. In particular, *A. amphigeneum* and *L. lallemandii* are Indo-Pacific, *A. armata* is Cosmopolitan, *B. hamifera* is Circumboreal, and *C. cylindracea* is Pantropical. Of these species, *A. armata* and *L. lallemandii* was already reported in the 1970s [16,17], while the other NIS were found for the first time in the present study.

**Figure 7:**
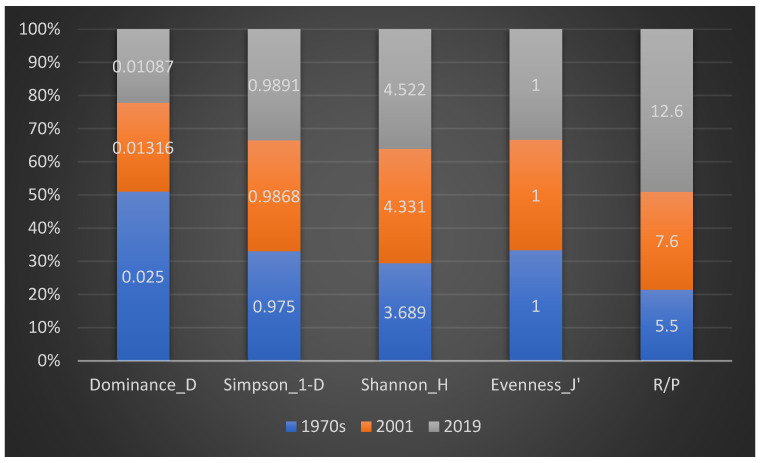
Values of Simpson’s Dominance index (D), Simpson’s index (1-D), Shannon’s index (H) Evenness index (J’) and R/P index in the coralligenous macroalgal assemblages of the 1970s, 2001, and 2019.

**Figure 8:**
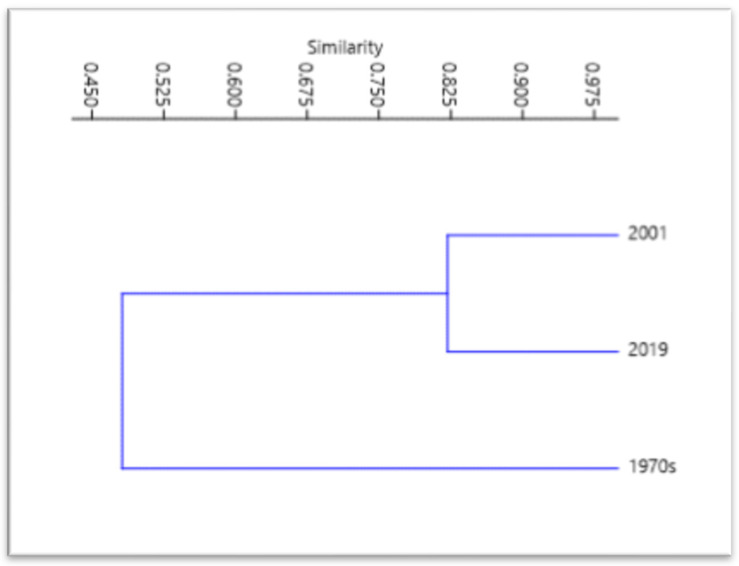
Dendrogram, calculated based on the Bray–Curtis similarity matrix, depicting mutual floristic similarities of the coralligenous floras investigated.

Regarding the biodiversity indices, Shannon’s (H) index and Simpson’s index (1-D) increased passing from the 1970s to 2019. Instead, Simpson’s Dominance index (D) decreased from the 1970s to 2019. The Evenness index (J’) remained stable from the 1970s to 2019, indicating that the species have always been well distributed in the coralligenous macroalgal assemblages (Figure 7). The Bray–Curtis similarity matrix displayed that the coralligenous flora of 2019 is linked to the flora of 2001 at a similarity level of 0.82, indicating that the two floras are very similar. On the contrary, the flora of the 1970s is linked to the flora of 2001 at a similarity level of 0.57, and the flora of 2019 at a similarity level of 0.40. Therefore, there is a high degree of affinity between the coralligenous floras of 2001 and 2019, while they present a low affinity with the coralligenous flora of the 1970s (Figure 8).

## 4. Discussion

**Figure 9:**
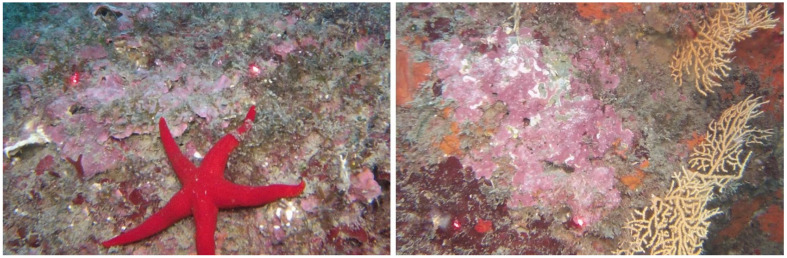
Two frames derived from ROV videos, showing a high coverage of encrusting Rhodophyta.

Through ROV surveys, it was observed that the coralligenous assemblages of the MPA Isole Ciclopi are represented by two facies, *Eunicella cavolinii* and *Lithophyllo-Halimedetum tunae* that are well-structured, especially regarding the encrusting Rhodophyta, which showed the highest percent cover (75–50%) among the main morphological groups/taxa (Figure 9). On the contrary, in the frames, it was noted that algal turf and NIS presented a percent cover of 25–5%. According to [21], the morphological groups/taxa characterizing the coralligenous present a different sensitivity level based on their biological traits. In particular, algal turf and NIS have a low sensitivity level, providing that they are stress-resistant. In fact, according to [26] turf-forming macroalgae are strong competitors compared to erect species because of their ability to quickly recover after disturbance. However, the higher percent cover of calcareous Rhodophyta, *Peyssonellia* sp. pl. and erect Rhodophyta than turf-forming and NIS could denote a good environmental status of the MPA’s coralligenous assemblages.

Regarding the floristic analysis, through the destructive samples, a total of 92 taxa was detected. Comparing the floristic lists of the present research and past studies [16,17,18], an increase of the local biodiversity was observed over approximately 50 years. Furthermore, the diversity indices (Simpson and Shannon) highlighted an increment of biodiversity, particularly from the 1970s to 2001, probably due to the establishment of the MPA in 1989. Moreover, Simpson’s Dominance index decreased from the 1970s to 2019, indicating a lower level of dominance of few species and the achievement of higher biodiversity. Nevertheless, throughout the years an increase in the R/P index was displayed. According to [27], this index increases when the stress and instability factors rise and it decreases under conditions of high structuring and environmental stability. As evidence of environmental instability, the number of Rhodophyta grows, while there is not a significant decrease in the number of Ochrophyta [28]. Similar variations were observed by comparing the coralligenous floras of the MPA over a period of about 50 years: the Rhodophyta significantly increased, while Ochrophyta remained almost stable. This occurrence could be related to reduced water transparency, due to the presence of suspended inert material which is assumed to favor the settlement of many sciaphilous Rhodophyta and/or members of this group with wide ecological valence [28]. Indeed, sedimentation is considered as one of the major threats for coralligenous habitat [10,11,29], since sediment can inhibit the rate of recruitment, growth and metabolic processes, limiting algal production [30]. During the examination of ROV frames, the presence of suspended material was often observed. This could be related to the presence of marine debris which is principally linked to the fishing activity in the MPA, as already remarked by [15].

Regarding the chorology, comparing the floristic lists obtained from the previous studies [16,17,18] with the current data, an increase of the Pantropical element and a decrease of the Mediterranean element, characteristic of deep subtidal environments, were noted. As highlighted by [31] this could denote an increase of warm-water species and a decreased proportion of cold-water species. Moreover, comparing the coralligenous floras, a gradual disappearance of the species of the order Fucales were detected from the 1970s to 2019. Both occurrences could be indicative of the seawater warming, as observed by [31,32] in Linosa and Pantelleria islands. Indeed, since the end of the 1980s, the Mediterranean Sea has suffered a regime shift in the atmospheric, hydrological, and ecological systems [33]. In particular, the Mediterranean Sea water warming favors, along with the interaction of multiple stressors factors (e.g., sedimentation, nutrient enrichment, and mechanical destruction), the establishment of NIS [34,35]. This warming not only stresses the native species, but also facilitates the arrival of other NIS, adding extra pressure on the ecosystem [36]. Indeed, comparing the floristic lists, an increment in the number of NIS was also displayed. In fact, in the 1970s two NIS (*A. armata* and *L. lallemandii*) were reported, in 2001 no new NIS was recorded, and in 2019 three new NIS (*A. amphigeneum*, *B. hamifera,* and *C. cylindracea*) were found, together with those previously observed. According to [37], since within the MPA there are three harbors, the main way of access of the NIS in the MPA could be related to shipping. However, as already reported by [37] and observed through ROV frames in this study, these NIS do not show a high coverage or an invasive attitude in the coralligenous of the MPA.

Overall, through the present study, an increase of the biodiversity in the MPA Isole Ciclopi was highlighted by comparing coralligenous floras of the 1970s, 2001, and 2019. Nevertheless, an increase of the NIS, warm-water species and Rhodophyta with wide ecological valence was also observed. The main causes of these variations in the coralligenous flora were traced back to reduced water transparency, maybe due to sedimentation, and a rise in the seawater temperature. Moreover, stressed ecosystems are more receptive to NIS, which in turn become further drivers of change [38]. If the MPAs cannot prevent climate change and invasive species [39,40], they can work towards the reduction of local anthropogenic stressors, enhancing the resilience of coastal marine habitats to global stressors [41]. Therefore, although the coralligenous assemblages of the MPA Isole Ciclopi appear to be well-structured, future studies will be necessary to continue monitoring this habitat to evaluate whether the MPA is effective in safeguarding this hotspot of biodiversity.

## Figures and Tables

**Table 1:** Summary table of ROV transects.

Transect	Depth	Length	Exposure	Latitude and Longitude Initial Point of the Transect/Final Point of the Transect
TLa	35 m	~200 m	East-South/East	37°33′41.50″ N 15°10′06.26″ E/37°33′34.82″ N 15°10′04.98″ E
TLb	33 m	~200 m	North/East-North	37°33′44.02″ N 15°10′01.85″ E/37°33′40.56″ N 15°10′08.73″ E
TLc	35 m	~200 m	South-South/West	37°33′25.09″ N 15°09′47.47″ E/37°33′24.79″ N 15°09′56.47″ E

**Table 2:** List of the macroalgal *taxa* found in the 1970s, 2001, and 2019 in the coralligenous habitat of the MPA Isole Ciclopi.

Chorology	Taxa	1970	2001	2019
	Rhodophyta			
M	*Acrodiscus vidovichii* (Meneghini) Zanardini	+		
IA	*Acrosorium ciliolatum* (Harvey) Kylin	+	+	+
IA	*Aglaothamnion tenuissimum* (Bonnemaison) Feldmann-Mazoyer	+	+	+
IA	*Aglaothamnion tripinnatum* (C. Agardh) Feldmann-Mazoyer	+	+	
P	*Amphiroa rubra* (Philippi) Woelkerling	+		
Abt	*Anotrichium barbatum* (C. Agardh) Nägeli		+	+
IP	*Antithamnion amphigeneum* A. Millar			+
IA	*Antithamnion cruciatum* (C. Agardh) Nägeli		+	+
IA	*Apoglossum ruscifolium* (Turner) J. Agardh	+	+	+
C	*Asparagopsis armata* Harvey (*Falkenbergia rufolanosa* stage)	+	+	+
CB	*Bonnemaisonia hamifera* Hariot (*Trailliella intricata* stage)			+
SC	*Caulacanthus ustulatus* (Turner) Kützing			+
C	*Centroceras clavulatum* (C. Agardh) Montagne		+	+
M	*Ceramium bertholdii* Funk	+	+	
SC	*Ceramium cimbricum* H.E. Petersen			+
SC	*Ceramium codii* (H. Richards) Feldmann-Mazoyer		+	+
IA	*Ceramium comptum* Børgesen		+	+
Ab	*Ceramium echionotum* J. Agardh			+
M	*Ceramium graecum* Lazaridou *et* Boudouresque			+
C	*Champia parvula* (C. Agardh) Harvey	+	+	+
M	*Contarinia peyssonneliaeformis* Zanardini		+	+
M	*Contarinia squamariae* (Meneghini) Denizot	+	+	+
Abt	*Croisettea requienii* (J. Agardh) M. J. Wynne	+		
SC	*Crouania attenuata* (C. Agardh) J. Agardh	+	+	+
IA	*Dasya ocellata* (Grateloup) Harvey		+	+
IA	*Eupogodon planus* (C. Agardh) Kützing		+	+
M	*Feldmannophycus rayssiae* (Feldmann et Feldmann-Mazoyer) H. Augier et Boudouresque	+	+	+
C	*Gelidium minusculum* (Weber Bosse) R.E. Norris		+	+
C	*Gelidium pusillum* (Stackhouse) Le Jolis		+	+
M	*Gloiocladia furcata* (C. Agardh) J. Agardh		+	+
IA	*Gloiocladia repens* (C.Agardh) N.Sánchez & Rodríguez-Prieto	+	+	+
IA	*Griffithsia opuntioides* J. Agardh			+
Abt	*Halydictyon mirabile* Zanardini		+	+
SC	*Halymenia floresia* (Clemente) C.Agardh			+
Abt	*Haraldia lenormandii* (Derbès & Solier) Feldmann		+	+
P	*Herposiphonia secunda* (C. Agardh) Ambronn		+	+
P	*Herposiphonia tenella* (C. Agardh) Ambronn		+	+
IA	*Heterosiphonia crispella* (C. Agardh) M.J. Wynne		+	+
C	*Hydrolithon boreale* (Foslie) Y.M. Chamberlain		+	+
IA	*Hydrolithon cruciatum* (Bressan) Y.M. Chamberlain			+
C	*Hydrolithon farinosum* (J.V. Lamouroux) Penrose et Y.M. Chamberlain v. *farinosum*	+	+	+
Ab	*Hypoglossum hypoglossoides* (Stackhouse) Collins et Hervey	+	+	+
Ab	*Irvinea boergesenii* (Feldmann) R.J. Wilkes, L.M. McIvor et Guiry			+
SC	*Jania adhaerens* J.V. Lamouroux		+	+
C	*Jania rubens* (Linnaeus) J.V. Lamouroux		+	+
IAtf	*Jania virgata* (Zanardini) Montagne	+		
Abt	*Laurencia chondrioides* Børgesen			+
C	*Laurencia obtusa* (Hudson) J.V. Lamouroux		+	+
P	*Lejolisia mediterranea* Bornet			+
IA	*Lithophyllum pustulatum* (J.V. Lamouroux) Foslie		+	+
IA	*Lithophyllum stictaeforme* (Areschoug) Hauck	+	+	+
M	*Lomentaria chylocladiella* Funk		+	+
M	*Lomentaria claviformis* Ercegovic	+	+	+
M	*Lomentaria ercegovicii* Verlaque, Boudouresque, Meinesz, Giraud & Marcot Coqueugniot		+	+
IP	*Lophocladia lallemandii* (Montagne) F. Schmitz	+	+	+
Abt	*Meredithia microphylla* (J. Agardh) J. Agardh		+	+
M	*Mesophyllum expansum* (Philippi) Cabioch & M.L.Mendoza	+	+	
M	*Mesophyllum macroblastum* (Foslie) W.H. Adey			+
M	*Metapeyssonnelia feldmanni* Boudouresque, Coppejans et Marcot			+
IA	*Nitophyllum punctatum* (Stackhouse) Greville		+	+
M	*Nitophyllum tristromaticum* J.J. Rodriguez y Femenìas ex Mazza (i.s.) *	+	+	+
P	*Peyssonnelia bornetii* Boudouresque et Denizot		+	+
M	*Peyssonnelia crispata* Boudouresque et Denizot		+	+
IA	*Peyssonnelia dubyi* P. et H. Crouan		+	+
Ab	*Peyssonnelia harveyana* P. et H. Crouan ex J. Agardh			+
SC	*Peyssonnelia heteromorpha* (Zanardini) Athanasiadis		+	+
M	*Peyssonnelia magna* Ercegovic		+	+
Abt	*Peyssonnelia rosa-marina* Boudouresque et Denizot	+	+	+
P	*Peyssonnelia rubra* (Greville) J. Agardh	+	+	+
M	*Peyssonnelia squamaria* (S.G. Gmelin) Decaisne	+	+	
A	*Phymatolithon lamii* (Me. Lemoine) Y.M. Chamberlain			+
SC	*Plocamium cartilagineum* (Linnaeus) P.S. Dixon	+	+	+
C	*Pneophyllum fragile* Kützing		+	+
M	*Polysiphonia atra* Zanardini	+		
M	*Polysiphonia perforans* Cormaci, G. Furnari, Pizzuto et Serio			+
P	*Polysiphonia scopulorum* Harvey		+	+
Ab	*Polysiphonia subulata* (Ducluzeau) Kützing	+		
SC	*Pterothamnion crispum* (Ducluzeau) Nägeli	+	+	+
SC	*Pterothamnion plumula* (J. Ellis) Nägeli			+
IA	*Ptilothamnion pluma* (Dillwyn) Thuret	+	+	+
M	*Rhodymenia leptofaucheoides* P.Huvé & H.Huvé			+
Abt	*Rhodymenia pseudopalmata* (J.V. Lamouroux) P.C. Silva		+	+
M	*Sebdenia monardiana* (Montagne) Berthold	+	+	+
Ab	*Sphaerococcus coronopifolius* Stackhouse	+	+	+
C	*Stylonema alsidii* (Zanardini) K.M. Drew	+	+	
A	*Vickersia baccata (J. Agardh) Karsakoff*			+
P	*Wrangelia penicillata* (C. Agardh) C. Agardh		+	+
	Ochrophyta			
M	*Cystoseira dubia* Valiante	+	+	
C	*Dictyopteris polypodioides* (A.P. De Candolle) J.V. Lamouroux	+	+	
SC	*Dictyota dichotoma* (Hudson) J.V. Lamouroux v. *intricata* (C. Agardh) Greville	+	+	+
SC	*Halopteris filicina* (Grateloup) Kützing	+	+	+
P	*Sargassum acinarium* (Linnaeus) Setchell	+		
SC	*Sphacelaria cirrosa* (Roth) C. Agardh		+	+
Ab	*Sphacelaria plumula* Zanardini	+	+	+
AP	*Zanardinia typus* (Nardo) G. Furnari		+	+
IA	*Zonaria tournefortii* (J.V. Lamoroux) Montagne		+	+
	**Chlorophyta**			
C	*Bryopsis hypnoides* J.V. Lamouroux		+	+
P	*Caulerpa cylindracea* Sonder			+
Abt	*Codium bursa* (Linnaeus) C. Agardh		+	+
At	*Flabellia petiolata* (Turra) Nizamuddin	+	+	+
P	*Halimeda tuna* (J. Ellis et Solander) J.V. Lamoroux		+	+
IA	*Lychaete pellucida* (Hudson) M.J. Wynne			+
SC	*Ostreobium quekettii* Bornet et Flahault			+
APt	*Palmophyllum crassum* (Naccari) Rabenhorst		+	+
SC	*Ulvella lens* P. et H. Crouan		+	+
P	*Valonia macrophisa* Kützing		+	+

Species are listed in alphabetical order within each major taxonomic group. Each species is preceded by the indication of the phytogeographic elements. For their indication the following abbreviations have been used: A = Atlantic; Ab = boreo-Atlantic; Abt = boreo-tropical Atlantic; At = tropical Atlantic; AP = Atlanto-Pacific; Apt = Atlanto-Pacific tropical; C = Cosmopolite; CB = Circumboreal; IA = Indo-Atlantic; IP = Indo-Pacific; M = Mediterranean; P = Pantropical; SC = Sub-Cosmopolitan. For the elaboration of the chorology in the graphics, A refers to A + Ab + Abt + At + AP + Apt + IA, C refers to C + SC, CB refers to CB, IP refers to IP, M refers to M, P refers to P. * (i.s.) is from the Latin incertae sedis.

**Table 3:** Percent cover (and corresponding degrees of the Braun-Blanquet’s scale) of the main morphological groups/taxa characterizing the coralligenous assemblage of the MPA.

Groups/Taxa	Percent Cover	Grades of Braun-Blanquet’s Scale
Calcareous Rhodopyta	75–50%	4
*Peyssonellia* sp. pl.	50–25%	3
Erect corticated Rhodophyta	50–25%	3
Algal turf	25–5%	2
NIS	25–5%	2
*Palmophyllum crassum*	5–1%	1
*Halimeda tuna*	<1%	+
Flattened corticated Rhodophyta	<1%	+

**Table 4:** Variations in the number of species, Rhodophyta, Ochrophyta, and Chlorophyta observed from the 1970s to 2019 in the coralligenous flora of the MPA.

	Species Reported in 2001	Species No Longer Found in 2001	Species Reported in 2019	Species No Longer Found in 2019	Species Present in All Sampling Periods
N. species	43	7	23	7	26
Rh	34	6	20	6	22
Oc	3	1	0	1	3
Ch	6	0	3	0	1

**Table 5:** Variations in the number of Atlantic, Cosmopolitan, Circumboreal, Indo-Pacific, Mediterranean, and Pantropical species observed from the 1970s to 2019 in the coralligenous flora of the MPA.

	Species REPORTED in 2001	Species No Longer Found in 2001	Species Reported in 2019	Species No Longer Found in 2019	Species Present in All Sampling Periods
Atlantic	17	3	9	1	11
Cosmopolitan	13	0	5	2	8
Circumboreal	0	0	1	0	0
Indo-Pacific	0	0	1	0	1
Mediterranean	6	2	5	4	5
Pantropical	7	2	2	0	1
